# Feasibility of a multicomponent cognitive behavioral intervention for fear of falling after hip fracture: process evaluation of the FIT-HIP intervention

**DOI:** 10.1186/s12877-021-02170-5

**Published:** 2021-04-01

**Authors:** Maaike N. Scheffers-Barnhoorn, Monica van Eijk, Jos M. G. A. Schols, Romke van Balen, Gertrudis I. J. M. Kempen, Wilco P. Achterberg, Jolanda C. M. van Haastregt

**Affiliations:** 1 Department of Public Health and Primary Care, Leiden University Medical Center, Postbox 9600, Leiden, 2300 RC The Netherlands; 2 Department of Health Services Research and Care and Public Health Research Institute (CAPHRI), Maastricht University, Maastricht, The Netherlands; 3 Department of Family Medicine and Care and Public Health Research Institute (CAPHRI), Maastricht University, Maastricht, The Netherlands

**Keywords:** Process evaluation, Feasibility, Fear of falling, Hip fracture, Cognitive behavioral intervention, Geriatric rehabilitation

## Abstract

**Background:**

This study describes the process evaluation of an intervention developed to reduce fear of falling (FoF) after hip fracture, within an inpatient geriatric rehabilitation setting. This ‘FIT-HIP intervention’ is a multicomponent cognitive behavioral intervention, conducted by physiotherapists and embedded in usual care in geriatric rehabilitation in the Netherlands. A previous study (cluster randomized controlled trial) showed no beneficial effects of this intervention when compared to usual care. The aim of this study was to gain insight into factors related to the intervention process that may have influenced the effectiveness of the intervention.

**Methods:**

This process evaluation was conducted using an observational prospective study design. Based on quantitative and qualitative data derived from session logs, evaluation questionnaires and interviews, we addressed: 1] recruitment and reach; 2] performance according to protocol; 3] patients’ adherence; and 4] opinions of patients and facilitators on the intervention. Participants in this study were: a) patients from 6 geriatric rehabilitation units, who were invited to participate in the intervention (39 adults aged ≥65 years with hip fracture and FoF) and; b) intervention facilitators (14 physiotherapists and 8 psychologists who provide coaching to the physiotherapists).

**Results:**

Thirty-six patients completed the intervention during inpatient geriatric rehabilitation. Apart from cognitive restructuring and telephonic booster (which was not provided to all patients), the intervention was performed to a fair degree in accordance with protocol. Patients’ adherence to the intervention was very good, and patients rated the intervention positively (average 8.1 on a scale 0–10). Although most facilitators considered the intervention feasible, a limited level of FoF (possibly related to timing of intervention), and physiotherapists’ limited experience with cognitive restructuring were identified as important barriers to performing the intervention according to protocol.

**Conclusions:**

The FIT-HIP intervention was only partly feasible, which may explain the lack of effectiveness in reducing FoF. To improve the intervention’s feasibility, we recommend selecting patients with *maladaptive* FoF (i.e. leading to activity restriction), being more flexible in the timing of the intervention, and providing more support to the physiotherapists in conducting cognitive restructuring.

**Trial registration:**

Netherlands Trial Register: NTR5695 (7 March 2016).

**Supplementary Information:**

The online version contains supplementary material available at 10.1186/s12877-021-02170-5.

## Background

Many older adults who have sustained a hip fracture will go through an extensive and generally challenging process of rehabilitation [1, 2]. During this recovery process, a substantial number of patients will experience concerns about falling (once) again [3, 4]. This fear of falling (FoF), is defined as ‘*a lasting concern about falling that leads to an individual avoiding activities that he/she remains capable of performing’.* [5] Prevalence rates of up to 63% have been reported for FoF in inpatient geriatric rehabilitation after hip fracture [4]. As a consequence of the activity restriction associated with FoF, deterioration in physical functioning and a decline in social participation and quality of life can occur [3, 6]. FoF may even have more effect on functional recovery after fracture than pain and depression [7]. Hence, FoF appears to be an important risk factor for impaired recovery [3, 8, 9], which could possibly be addressed by treatment.

Patients with a recent hip fracture differ from the general population of community-dwelling older adults in that they experience a sudden impairment of their gait function and consequently become dependent in (basic) activities of daily living [2]. In the Netherlands, approximately half of all older patients with a hip fracture follow an inpatient multidisciplinary rehabilitation program after surgical repair of the fracture. These ‘*geriatric rehabilitation*’ services are specialized in the medical care for frail older adults [10]. Therapy is aimed at optimizing the patient’s physical condition and restoring (gait) function [11]. Physical therapy focuses on training balance and muscle strength, and practicing activities of daily living [12]. At present there are no treatment programs aimed specifically at reducing FoF after a recent hip fracture. However, for community-dwelling older adults, various evidence-based interventions have been developed to reduce FoF [13–18]. Particularly the treatment programs that combine exercise with cognitive behavioral approaches have been found to be effective in reducing FoF [16–18]. In the Netherlands, two of these evidence-based programs using cognitive behavioral approaches have been nationally implemented (based on *‘A Matter of Balance’*) [15, 19, 20]. However, in their current format (community- or home-based), these established programs are not suitable for the therapeutic setting of inpatient geriatric rehabilitation. The cognitive behavioral approaches used in these programs were therefore adjusted to an individualized treatment program that fits the (physio) therapeutic setting within rehabilitation services. This *Fear of falling InTervention in HIP fracture geriatric rehabilitation* (FIT-HIP intervention) was designed to reduce FoF and consequently to improve functional outcome in inpatient geriatric rehabilitation after hip fracture [21]. However, a recent cluster randomized controlled trial evaluating the effects of the FIT-HIP intervention showed the program was not effective in reducing FoF or improving functional outcome after hip fracture [12].

The aim of this process evaluation therefore is to gain insight into factors that may have influenced the effectiveness of the intervention. Subsequently, findings from this study can provide insight into opportunities to improve both the intervention itself and its implementation in clinical practice. In this study we assessed the feasibility of the FIT-HIP intervention in clinical practice based on the following aspects of the intervention process: 1] recruitment and reach; 2] performance according to protocol (*dose delivered and fidelity*); 3] adherence (*dose received exposure*); and 4] opinion on the intervention provided by patients and facilitators (*dose received satisfaction and context*). These items are based on the framework of Saunders and colleagues [22, 23]. This model for process evaluation is frequently used within health care innovations and is based on the widely acknowledged principles of Steckler et al. (2002) [24].

## Methods

### Study design

This process evaluation has an observational prospective design, combining qualitative and quantitative research methods. It was conducted in conjunction with the cluster randomized controlled trial that evaluated effectiveness of the FIT-HIP intervention [12]. Ethical approval was provided by the Ethics Committee of the Leiden University Medical Center (LUMC) and the study was registered in the Netherlands Trial Register (NTR5695). Patients were recruited between March 2016 and January 2017 from 11 post-acute geriatric rehabilitation units in the Netherlands. For the present study we focused on the patients and intervention facilitators from the six units that were allocated to the FIT-HIP intervention.

### Intervention

The FIT-HIP intervention is an individualized, multicomponent intervention based on cognitive behavioral approaches. It aims to reduce FoF in inpatient geriatric rehabilitation after hip fracture. The intervention is conducted by physiotherapists from the participating units and is integrated in usual care in geriatric rehabilitation (i.e. physical therapy sessions). The following cognitive behavioral elements are embedded in the intervention: 1] guided exposure to feared activities; 2] cognitive restructuring; 3] psychoeducation; 4] relapse prevention (*Staying Active Plan* and telephonic booster); and 5] motivational interviewing. These elements are combined with regular exercise training in rehabilitation. The physiotherapists are counseled by psychologists (from participating units) during daily practice. This coaching is organized as (on-site) monthly meetings and interim consultation at the request of the physiotherapists.

The study protocol published previously [21] and Table 1 provide detailed information on the rationale and schedule of the various items within intervention. The intervention, which is integrated in the regular geriatric rehabilitation treatment, starts directly after admission and lasts for the duration of the inpatient rehabilitation (in general six to 7 weeks) [10]. First, patients have an intake interview with the physiotherapist, to assess which circumstances cause concerns of falling, and to determine treatment goals. Next, based on this information, the physiotherapist puts together a tailor-made treatment plan for the application of the guided exposure (i.e. the *FIT-HIP fear ladders*). Guided exposure is considered the core element of the FIT-HIP intervention and is applied within the regular physical therapy sessions as long as the FoF persists. Guided exposure may not be necessary in all sessions (in the event the FoF has subsided). Cognitive restructuring is also tailored to the patient’s needs. The frequency will depend on whether the patient has unrealistic thoughts and on the patient’s receptiveness to such an approach. Cognitive restructuring is practiced at least twice during the inpatient rehabilitation treatment (including a homework assignment) and can be repeated as needed. Psychoeducation is provided in the initial stage of rehabilitation (first 3 weeks) and in the final stage when discharge is being planned. In both stages the information is provided during at least one session. The psychoeducation in the final stage is integrated in the relapse prevention plan (i.e. *Staying Active Plan*), a reference book given to the patient at discharge. A topic list of the psychoeducation is provided in Additional file 1. The telephonic booster 6 weeks after discharge (one session) is the final element of the intervention. Motivational interviewing does not have a fixed schedule in the intervention, as it is applied by the physiotherapists during the entire FIT-HIP program, in order to assess and relate to the patient’s intrinsic and extrinsic motivation for rehabilitation.

**Table 1:** Overview of the FIT-HIP intervention

Element	Description
**Guided exposure –** *rationale*	Guided exposure is the graded and repeated exposure to situations that give rise to fear (of falling). As recurrent exposure to the feared situation or activity is performed under supervision and in a manner that is predictable and controllable, this leads to the positive experience that the fear gradually fades out as the activity is practiced more often. After the fear for this specific situation has subsided, the exposure can be extended to the ‘next level’, practicing the activity in a manner that leads to a greater level of fear (fear hierarchy for graded exposure). For fear of falling (FoF), the feared activities will be situations concerning physical activity. In the rehabilitation after hip fracture, this will predominantly be basic activities in daily living, such as transferring, standing and walking.
*Implementation in the FIT-HIP intervention*	In the FIT-HIP intervention the physiotherapist helps the participant assess situations that give rise to FoF (within the first week of admission to geriatric rehabilitation (GR)). For each ‘feared’ activity the physiotherapist and participant draft a fear hierarchy, designed as a ‘fear ladder’ (template example published in protocol) [21]. The FIT-HIP fear ladder consists of six ‘steps’, each step representing a functional goal. The functional goal describes in which manner the activity is practiced/performed. The goals are ranked with an increasing level of FoF as the activity gets more complex (or has to be performed with less assistance). The FIT-HIP fear ladders are the guiding principle for the multidisciplinary approach to apply guided exposure for all aspects of mobilization. The physiotherapist evaluates the fear ladders with the participant weekly and the fear ladders are revised on the basis of progress (reduction of FoF).
*Intervention provider(s)*^a^	Physiotherapists during physical therapy sessions. As applicable, by nursing staff when assisting patients in basic activities of daily living that give rise to FoF. Nursing staff assisting participants in practicing ‘fearful’ activities as ‘homework assignments’ after physical therapy.
*Schedule*	Incorporated in all physical therapy sessions (and nursing care activities) for the duration of inpatient multidisciplinary GR as long as FoF persists.
**Cognitive restructuring -** *rationale*	Thoughts (and associated beliefs) influence how a person feels and accordingly how a person appraises and responds to a situation. Excessive concern to fall (fear of falling) can be based on unrealistic thoughts and beliefs with regard to (risk of) falling. This excessive FoF may lead to avoidance of (physical) activity and consequently fortify the FoF. Cognitive restructuring is a technique used to explore thoughts and beliefs and therefore to identify, challenge and modify unrealistic thoughts. In the FIT-HIP intervention participants are coached to explore their thoughts concerning physical activity and fall risk. In doing so they are encouraged to identify maladaptive and unrealistic thoughts and in turn formulate and apply more realistic thoughts. The principle of (un) realistic thoughts is also incorporated into the relapse prevention plan (see below).
*Implementation in the FIT-HIP intervention*	Physiotherapists are trained to guide the participant in exploring their thoughts concerning physical activity and (risk of) falling. A worksheet is used to structure the process of cognitive restructuring and to provide the participant insight in this process (analyzing the situation and the associated thoughts, feelings, behavior and consequences and subsequently formulating more realistic thoughts).
*Intervention provider(s)*^a^	Physiotherapists. A psychologist is trained as a ‘buddy’ to coach the physiotherapists in these principles as when additional help is needed.
*Schedule*	During at least one physical therapy session the cognitive restructuring is applied and practiced with the participant. Subsequently, the participant is encouraged to fill in the worksheet as a ‘homework assignment’. This is reviewed and discussed during the next therapy session. These ‘key’ thoughts can briefly be recapitulated in situations when the FoF is noticeable in the physical therapy sessions. The process of cognitive restructuring can be repeated as needed (when the FoF persists).
**Psychoeducation -** *rationale and implementation in the FIT-HIP intervention*	The psycho-education is used to reinforce the various elements of the FIT-HIP intervention. In the initial phase of GR the participant receives information on anxiety, (consequences and treatment of) FoF and the rationale and background of guided exposure and cognitive restructuring. In the final phase of GR, when discharge home is being planned, the psycho-education focusses on home safety. The information on home safety is also processed in the relapse prevention plan (see below). For detailed information of the psychoeducation, see the topic list presented in Additional file 1
*Intervention provider(s)*^a^	Physiotherapists discuss the information with the participant.
*Schedule*	During at least two physical therapy sessions (one in the initial phase of rehabilitation; the other preceding the discharge home). As applicable, the psycho-education can additionally be incorporated in the therapy sessions, related to situations occurring during therapy (for example fall prevention).
**Relapse prevention -** *rationale*	The relapse prevention is aimed at helping the participant to anticipate and cope with relapse to FoF.
*Implementation in the FIT-HIP intervention*	In the FIT-HIP intervention the relapse prevention is designed to optimize the transition to predominantly independent living circumstances after discharge home. For this purpose, a ‘relapse prevention plan’ is composed together with the participant. This ‘*Staying Active Plan*’ aims at preparing the participant for challenging situations in which there is a risk for relapse to FoF and activity restriction. The ‘*Staying Active Plan*’ consists of (information on) 1. General home safety and fall prevention; 2. Individualized advice for safe ambulation and how to stay active; 3. Preventing, recognizing and dealing with a relapse (including notice of (mal)adaptive) thoughts). The information is discussed together with the participant and presented in writing as a reference book. In addition, a telephonic booster is conducted 6 weeks after discharge from GR. The telephonic booster is aimed at evaluating the FoF (and activity restriction). If necessary advice is given how to deal with FoF, in addition to the prior advice formulated in the ‘*Staying Active Plan*’.
*Intervention provider(s)*^a^	Both the ‘*Staying Active Plan*’ and telephonic booster are conducted by physiotherapists.
*Schedule*	During at least one physical therapy session during GR (‘*Staying Active Plan*’) and one telephonic booster session after discharge home.
**Motivational interviewing**	Physiotherapists are trained^a^ in motivational interviewing techniques to assist the participant in the process of behavior change. These techniques help the physiotherapist gain insight into the participant’s extrinsic and intrinsic motivation and explore which rehabilitation goals are important for the participant, in order to personalize treatment goals in the FIT-HIP intervention.

**Notes**: This table was published in *Journal of the American Medical Directors Association.* 2019;20 (7):857–865.e852. Scheffers-Barnhoorn MN, van Eijk M, van Haastregt JCM, et al. Effects of the FIT-HIP Intervention for Fear of Falling After Hip Fracture: A Cluster-Randomized Controlled Trial in Geriatric Rehabilitation. Copyright of Elsevier (2019)

^a^Physiotherapists received two training sessions (4 h each); psychologists one 4-h session (together with physiotherapists). Nursing staff was briefed on the background and rationale of guided exposure, in order to help them incorporate these principles in their work and to adhere to the ‘*FIT-HIP fear ladders*’ (45–60 min). Training was provided by the researcher (MSB) together with a cognitive behavioral therapist (BB; furthermore a health care psychologist and teacher). After training and start of the trial, the researcher (MSB) had regular telephonic sessions with the facilitators to discuss recruitment procedures and questions regarding the treatment protocol

### Participants

Patients were older adults (≥ 65 years) with fear of falling, admitted to inpatient geriatric rehabilitation following hip fracture. FoF was assessed using the following one-item question with a 5-point Likert scale, ‘*Are you concerned to fall*?’ (answer options: *never - almost never - sometimes - often - very often*). Eligible for participation were patients who reported concerns about falling at least ‘sometimes’. Exclusion criteria included conditions interfering with learnability [dementia; a score > 1 on the Hetero-anamnesis List Cognition (HAC) [25] (suggestive for premorbid cognitive disabilities); or major psychiatric disease]; furthermore, a pre-fracture Barthel index score < 15; pathologic hip fracture; life expectancy < 3 months; and insufficient mastery of the Dutch language. All patients provided written informed consent for participation in the study. Thirty-nine patients were included in the present study.

The intervention providers, from here forward entitled *facilitators*, were physiotherapists working in the participating intervention units (two per unit), and psychologists. The physiotherapists were actively engaged in the multidisciplinary geriatric rehabilitation team and had experience in the field of (orthopedic) rehabilitation for frail older adults. One psychologist from each unit was involved for the on-site coaching of physiotherapists. Most participating units were specialized in orthopedic rehabilitation and the patient volume of these units varied from 19 to 34. Initially facilitators from six units were trained, but due to a limited inclusion rate after 4 months, we included an additional unit (affiliated to one of the participating units). In total, 14 physiotherapists (12 female) and eight psychologists (all female) were involved in the FIT-HIP program, and all were trained to perform the FIT-HIP intervention. For training details: see Table 1.

### Data collection

Table 2 presents an overview of the measurement instruments used to assess information for this process evaluation. Patients received a self-administered evaluation questionnaire at discharge from geriatric rehabilitation; and again at three and 6 months after discharge. We applied purposive sampling for the qualitative interviews with patients [26], and aimed to conduct interviews with a selection of patients from all participating units and representing both sexes, until data saturation occurred. Patients were approached by telephone for the interviews. Physiotherapists were asked to fill in session logs for all therapy sessions, providing information on attendance, therapy content (which FIT-HIP elements were performed), reasons to deviate from protocol and the duration of therapy. Adherence was assessed using the *Pittsburg Rehabilitation Participation Scale* (PPRS) to score participants’ active engagement during therapy. The PPRS is a 6-point Likert scale ranging from ‘*none*’ (patient refused therapy) to ‘*excellent*’. The physiotherapists were approached for a semi-structured site-specific group interview, and psychologists for a telephone interview. They also received an evaluation questionnaire. As physicians and nursing staff are also involved in the general rehabilitation process, they were approached to fill in a short evaluation questionnaire (five questions), to assess the extent to which they had been informed of or involved in the patients’ FIT-HIP treatment.

**Table 2:** Outcome measures and associated measurement instruments used for the FIT-HIP process evaluation

	Registration forms		Evaluation questionnaires			Interviews		Other
	Physiotherapy session log	Telephonic booster log	Patient (T1,2,3)^a^	Facilitator^b^	GR team^c^	Patient	Facilitator^b^	Log researcher^d^
**Recruitment**								
Barriers to recruitment							X	X
Maintaining patient engagement							X	X
**Performance according to protocol**								
Intervention items conducted	X	X						
Reasons to deviate from protocol	X						X	
**Patient adherence**								
Active participation during physical therapy	X							
Reasons for not attending physical therapy	X							
Adherence to homework			X					
Use of *‘Staying Active Plan’*			X					
**Opinion on the intervention**								
Overall opinion on the intervention			X			X	X	
Opinion of the value of the intervention (benefit)			X	X	X	X	X	
Perceived burden of the intervention			X			X		
Feasibility to perform the intervention				X			X	
Barriers to performing or implementing the intervention				X			X	X
Suggestion for improvement of the intervention			X	X	X	X	X	X

**Notes**: *GR* Inpatient Geriatric Rehabilitation. ^a^T1 = at discharge from GR, T2 = 3 months after discharge from GR, T3 = 6 months after discharge from GR; ^b^Facilitator = physiotherapist and psychologist; ^c^GR team = elderly care physician and nursing staff. ^d^Log researcher = log of additional data recorded by research (assistants), including reasons for dropout and information from informal evaluations with facilitators during study

Interviews performed by author MSB (clinician - trainee elderly care physician + PhD student, not involved in clinical care for the participants of the study). Setting: patient interviews in participant’s home. Facilitator interviews in clinic. Duration interviews: 1 h

Interviews were conducted after the six-month follow-up. They were performed by the author MSB and recorded on audiotape (with the exception of the telephone interviews).

### Data analysis

Quantitative data from the questionnaires and the session logs was analyzed by means of descriptive statistics using IBM SPSS Statistics version 23. The qualitative data from open-end questions in the questionnaires, session logs and the interviews, were transcribed and categorized based on content by author MS. Telephone interviews were summarized and categorized.

## Results

### Recruitment, reach and response

**Fig. 1:**
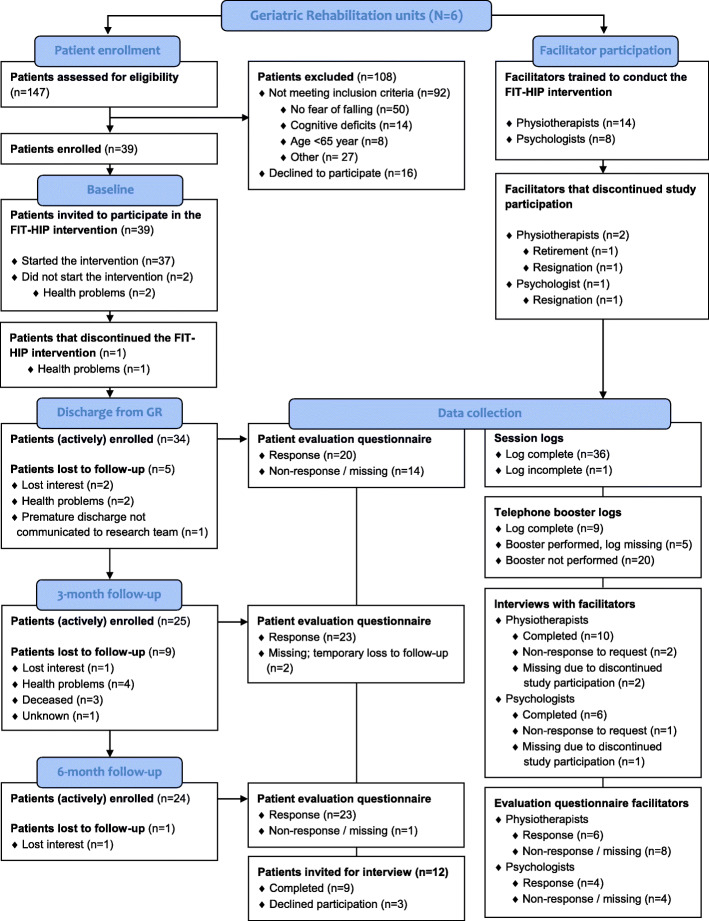
Flow diagram of the FIT-HIP process-evaluation

Enrollment of patients per unit varied from 1 to 11 (Additional file 2). Thirty-nine patients were assigned to the FIT-HIP intervention, 34 of whom were female (87.2%). Age varied from 65 to 98 years (mean: 83.7 ± 7.3) and the majority lived alone prior to the fracture (*n* = 27; 69.2%). At baseline one-third of the patients experienced concerns to fall (very) often, and the mean FES-I score (*Falls Efficacy Scale-International*) was 33.9 (SD:9.9); see also Additional file 3. The flow chart presented in Fig. 1 shows recruitment, reach and response for both patients and facilitators. The timing of enrollment for the study (first week of rehabilitation) was regularly experienced as inconvenient by patients, as it was difficult for them to anticipate and oversee both the rehabilitation (treatment program) and participation in the study. The main challenge for maintaining patient engagement in the study was poor health. Thirty-six of the 39 patients completed the intervention during inpatient rehabilitation. Two patients did not receive the intervention and one withdrew from treatment in the final stage of rehabilitation due to health problems.

Based on patients that were actively enrolled in the study at the various assessments, the response rate for the patients’ evaluation questionnaires was 58.8% (*n* = 20) at discharge; and 92% (*n* = 23) and 95.8% (n = 23) at three and 6 months follow-up. We conducted interviews with nine patients; three patients declined to be interviewed. All units were represented within the interviews, with the exception of unit 4 (*n* = 1 patient enrolled; Additional file 2). We excluded one session log from data analysis, as data were largely missing.

Two physiotherapists and one psychologist discontinued participation (Fig. 1). One of these physiotherapists had treated one patient according to the FIT-HIP intervention, the other had no FIT-HIP patients. Ten of the 14 physiotherapists and six of the seven psychologists participated in the interviews. Response rates for health care professionals’ evaluation questionnaires were: *N* = 6 for physiotherapists (42.9%; representing four units); *N* = 4 for psychologists (50.0%; representing three units); N = 4 for physicians (44.4%; representing three units) and N = 4 for nursing staff (representing two units).

### Performance according to protocol

The FIT-HIP intervention was conducted during inpatient geriatric rehabilitation and in our study the length of stay varied from 21 to 98 days (median: 42). From study inclusion until discharge, patients on average received 30.7 physiotherapy sessions (range: 8–105), accounting for 15.7 h of physiotherapy (range: 3.9–52.5).

Table 3 provides an overview of the dose delivered per FIT-HIP intervention element. The FIT-HIP intake was carried out for all patients. Guided exposure, the key element of the intervention, was delivered to 97.2% of the patients (*n* = 35). Lack of FoF after enrollment was the reason for not using guided exposure (*n* = 1). On average, guided exposure was incorporated in 56.6% of all physiotherapy sessions (ranging from 5 to 100%; tailored to patient’s needs and response to treatment). Cognitive restructuring was performed according to protocol less frequently; 26 patients (72.2%) had this element within their treatment program. On average cognitive restructuring was incorporated in 3.5 ± 1.9 sessions. Eighteen patients (50.0%) received homework assignment(s) for cognitive restructuring. With regard to reasons for deviating from protocol for cognitive restructuring, lack of FoF was mentioned for three patients, and for the remaining seven patients the reason was unknown. The telephonic booster was carried out for 38.9% of the patients (*n* = 14; of which *n* = 9 were registered in booster log), resulting in this being the intervention element that was most frequently not performed according to protocol. Facilitators from unit 3 forgot to perform the booster (*n* = 11 patients), one patient was repeatedly not available, and for the remaining patients who did not receive the booster, the reason was unknown.

**Table 3:** Performance according to protocol

	Patients from all units (***n*** = 36)^**a**^		
	n	%	Min-max
**FIT-HIP intake**			
Number of patients who received the FIT-HIP intake	36	100	^b^
**Guided exposure**			
Number of patients with ≥1 session(s) of guided exposure	35	97.2	^b^
Mean number of sessions with guided exposure per patient^c^; mean (SD)	18.9 (18.3)	^b^	1–95
Percentage of therapy sessions with guided exposure^c^; mean (SD)	^b^	56.6 (28.3)	5–100
**Psychoeducation**			
Number of patients with ≥1 session(s) of psychoeducation within the first 3 weeks of study participation	34	94.4	^b^
Mean number of sessions with psychoeducation within the first 3 weeks of study participation per patient^c^; mean (SD)	1.9 (1.3)	^b^	1–7
**Cognitive restructuring (homework)**			
Number of patients with ≥1 session(s) with cognitive restructuring	26	72.2	^b^
Mean number of sessions with cognitive restructuring per patient^c^; mean (SD)	3.5 (1.9)	^b^	1–8
Number of patients who received ≥1 homework assignment for cognitive restructuring	18	50.0	^b^
Mean number of sessions registered for cognitive restructuring homework per patient^c^; mean (SD)	1.8 (1.2)	^b^	1–6
**Staying Active Plan**			
Number of patients who received a *Staying Active Plan*	34	94.4	^b^
Mean number of sessions registered for the *Staying Active Plan* per patient ^c^; mean (SD)	2.0 (1.0)	^b^	1–4
**Telephonic booster**			
Number of patients who received the telephonic booster after discharge	14	38.9	^b^

**Notes:** ^a^All patients who (in part) received the FIT-HIP intervention (*n* = 37); data missing from *n* = 1 patient. ^b^Not applicable. ^c^Based on patients who have received that element of the FIT-HIP intervention

Regarding the coaching of physiotherapists provided by psychologists, interviews revealed that the frequency of meetings decreased over time. At the start of the study, meetings were initiated and the intervention protocol was reviewed again within the team. However, during the course of the study there were few consultation requests from the physiotherapists and consequently the meetings did not take place each month.

### Adherence

Based on the PRPS, active participation during the intervention sessions was very good to excellent for the majority of patients (56%; *n* = 20). One patient’s participation was rated as ‘fair’, the remaining 15 (41.2%) as ‘good’. Patients reported their adherence to homework (including physical exercises) as follows: during rehabilitation they performed their homework *‘sometimes’* (11.1%; *n* = 2), *‘most of the time’* (55.6%; *n* = 10) or *‘always’* (33.3%; *n* = 6). Time spent on homework varied from 30 to 420 min per week. Three months post-discharge eight patients (42.1%) had *‘never’* used the *Staying Active Plan;* three patients (15.8%) *‘seldom or sometimes’* and eight patients *‘most of the time’*. The reported adherence for the *Staying Active Plan* at 6 months was comparable.

### Opinion on the intervention

#### Patient opinions

In general, patients had a positive opinion about the treatment provided by physiotherapists and rated this with a mean of 8.1 (scale 0–10 with higher scores indicating a more favorable opinion) (range 6–10; *n* = 19). Ninety percent of the patients (*n* = 18) evaluated quality of the facilitators as being (very) good. A large majority of the patients would recommend this treatment for fear of falling to other patients (88.2%; *n* = 15). In general, the perceived burden of the physical effort during physiotherapy was rated as being ‘*just right’* (65.0%; *n* = 13), yet 25.0% experienced it as *‘too much’.* Using a 5-point Likert scale we assessed the perceived benefit of the intervention. At discharge from rehabilitation, half of the patients reported that the intervention was (very) helpful to reduce fear of falling and none reported having experienced no benefit from the intervention. The reported benefit after discharge decreased to 39.1% (*n* = 9) at 3 months, and 33.4% (*n* = 6) at 6 months. Patients reported most benefit from the *Staying Active Plan* (75.1%), guided exposure (62.5%) and psychoeducation (55.6%) (Table 4). After discharge, the reported benefit of the *Staying Active Plan* decreased to 35.7 and 36.4% after three and 6 months. The telephonic booster was considered least beneficial.

**Table 4:** Patients’ perceived benefit of the FIT-HIP intervention^b^

	Assessment		
	Discharge	3 months follow-up	6 months follow-up
This intervention item was (very) helpful to reduce the fear of falling^**a**^	n (%)	n (%)	n (%)
Psychoeducation (*n* = 18)	10 (55.6)	^b^	^b^
Guided exposure (*n* = 16)	10 (62.5)	^b^	^b^
Cognitive restructuring (*n* = 16)	7 (43.8)	^b^	^b^
Cognitive restructuring homework (*n* = 15)	6 (40.0)	^b^	^b^
Staying Active Plan (in general) (*n* = 16 / *n* = 14 / *n* = 11)	12 (75.1)	5 (35.7)	4 (36.4)
Telephonic booster (*n* = 11)	^b^	1 (9.1)	^b^

**Notes**: ^a^Based on a 5-point Likert scale with answer categories: *not at all; barely; a little; a lot; very much*. The last two answer categories (a lot; very much) describe that the intervention was (very) helpful to reduce fear of falling. ^b^Not applicable

Interviews showed the patients were positive about the physiotherapists. The patient-therapist relationship was mentioned as an important facilitator for recovery. Patients specified the following key factors within this patient-therapist relationship: 1] trust in the competence of the therapist; 2] calm and supportive personality of the therapist; 3] personal attention for the patient during therapy; and 4] the continuity in treatment - provided by that specific therapist. The fact that therapy was provided on a daily basis - sometimes multiple sessions - was helpful to (re) gain self-confidence. Additionally, patients experienced that having other patients as a reference during group sessions was supportive for recovery.

#### Care professionals’ opinions

The majority of the physiotherapists (70%, *n* = 7, representing four units) had a favorable opinion of the intervention and stated it was a good intervention for the treatment of FoF. These seven physiotherapists mentioned that intervention items such as psychoeducation, guided exposure and to some extent cognitive restructuring are already part of their (physiotherapy) treatment, but receive more attention and are offered in a more structured manner because of the intervention. Preferences for type of cognitive behavioral approach did, however, differ among these physiotherapists (guided exposure *n* = 4; cognitive restructuring *n* = 1; use of guided exposure or cognitive restructuring tailored to patient’s response to these approaches *n* = 2). Both physiotherapists and psychologists mentioned that this cognitive restructuring can be challenging for physiotherapists, depending on prior experience with psychosocial interventions. All facilitators questioned to what extent patients would use the *Staying Active Plan* after discharge.

For the physiotherapists with a less favorable opinion of the intervention, time constraints were an important barrier to performing the intervention according to protocol. They felt that treatment of fear (of falling) was more appropriate for psychologists and doubted the added value of the guided exposure principles over current usual care. Physiotherapists with positive attitudes toward the intervention (*n* = 7), on the other hand, did not perceive time as a barrier to implementing the intervention (for future purposes). Although (mild) cognitive impairment was regularly observed in the study population, this was usually not perceived to be a barrier to applying treatment principles. Additional file 4 presents an overview of all challenges, barriers and suggestions for improvement that were mentioned in this process evaluation; the main suggestions are highlighted below.

#### Suggestions for improvement

First, physiotherapists observed that after enrollment, the level of FoF among patients appeared to be limited, which consequently hindered the execution of the intervention according to protocol. To improve the efficiency and feasibility of the intervention on that account, it may be helpful to reconsider the selection of the target group (i.e. screening), and initiate treatment at a later stage of geriatric rehabilitation (i.e. if the FoF persists). Second, physiotherapists indicated that having more flexibility to tailor the treatment protocol to the individual patient would be helpful. In their experience, some patients were more receptive to guided exposure and others to cognitive restructuring. Hence, it would be useful to choose the most appropriate element for each individual patient, for example based on their treatment response and anxiety trait(s).

The third suggestion was to intensify the collaboration (and coaching function) between psychologists and physiotherapists, specifically with regard to cognitive restructuring. Although most physiotherapists felt they were capable of (partly) performing cognitive restructuring (as appropriate, with additional training and experience), they suggested it would be helpful if the psychologist routinely observed a physiotherapy session (for example once every week or 2 weeks). This would provide the opportunity to give additional advice to the physiotherapist, but also to monitor whether additional (psychological) treatment is required. To promote an interdisciplinary approach to addressing FoF, it was also recommended to train nursing staff in early recognition of FoF.

## Discussion

This study assessed the feasibility of a multicomponent cognitive behavioral intervention for FoF after hip fracture, integrated in usual care in inpatient rehabilitation. To a fair degree the intervention was performed according to protocol, but cognitive restructuring and the telephonic booster were not provided to all patients. Patients rated the intervention positively and half of them reported that the intervention was (very) helpful in reducing FoF. Most facilitators were positive about the intervention and considered it feasible. However, this study also identified barriers that may have affected this feasibility, and these should be addressed to improve the intervention. Two important barriers were the limited level of FoF after enrollment (possibly related to timing of the intervention), and the fact that physiotherapists, having limited experience with such approaches, perceived cognitive restructuring as challenging.

A considerable body of evidence demonstrates that programs based on cognitive behavioral approaches (preferably combined with physical exercise) are effective to reduce FoF in older adults with fall risk [16–18, 27]. However, despite the benefit perceived by patients, the FIT-HIP intervention was not effective in reducing FoF when compared to usual care [12]. It is therefore crucial to reflect on the intervention process, in particular cognitive restructuring as this was not administered to all patients and was considered the most challenging element for facilitators. First, the dose of cognitive restructuring within the intervention does not differ significantly from other programs [14, 28, 29], and this does not explain the absence of effect. However, in our study fewer patients received cognitive restructuring according to protocol (72.2% in the FIT-HIP study versus 83.4% in the home-based program for FoF in community-dwelling older adults) [30]. This may have contributed to the lack of effectiveness.

The fact that cognitive restructuring is perceived as challenging does not by definition imply it is not feasible in practice or not suitable for frail older adults. Literature on nurse-led programs for FoF in community-dwelling older adults confirms the finding that cognitive restructuring can be challenging for facilitators and participants, yet these programs - despite the perceived difficulties - proved to be effective [14, 15, 20]. Regarding the appropriateness of cognitive restructuring for frail older adults, facilitators in our study acknowledged that even in cases of mild cognitive impairment, this approach still had potential short-term effects (during the therapy session), enhancing the rehabilitation process.

In a broader perspective, we could question whether it is appropriate for physiotherapists to apply cognitive restructuring. In the past years, interest in incorporating a biopsychosocial approach to physiotherapy practice to enhance the rehabilitation process has increased [31]. Research illustrates that overall, physiotherapists have positive attitudes and beliefs regarding psychosocial interventions [31]. Common barriers to implementation of psychosocial interventions in clinical practice include lack of knowledge, time constraints (including the perceived need to prioritize physical care) and the scope of practice (role clarity and public perceptions of traditional physiotherapist role) [31, 32]. These factors were also identified in our study, but rather than the lack of knowledge, the facilitators mentioned a desire for more experience. The current literature concerning psychosocial interventions with physiotherapists as facilitators recommends that, in order to ensure treatment fidelity, psychologists should provide comprehensive training and mentoring to the physiotherapists, including performance feedback [32, 33]. Effectiveness of such an approach is supported by a recent study that showed positive effects of a physiotherapist-led in-home intervention to reduce FoF and activity avoidance, including cognitive restructuring and exposure therapy, in community-dwelling older adults [18]. The physiotherapists received weekly supervision by a psychologist, based on video tapes of the therapy sessions. Likewise, the *‘Step by Step intervention’* aimed at reducing FoF after hip- or pelvic fracture, performed by physiotherapists who received weekly supervision by clinical psychologists, also had favorable effects on reducing FoF [27]. In our intervention protocol the supervision by psychologists was limited to monthly team meetings and individual coaching on request. In practice this supervision occurred less frequently. This is therefore an area of attention for the future.

Reflecting on the *therapy intensity* in our intervention, thus comparing the individual intervention items to various effective multi-component interventions for FoF, is not straightforward, as this is not always described in detail in the available literature. Also, tailoring of the intervention to the specific needs of the patients can complicate insight in the therapy intensity. The core element of the FIT-HIP intervention is guided exposure to feared activities, which is integrated in most of the therapy sessions. In other intervention programs this element was generally limited to one or two therapy sessions [28, 33]. Only the ABLE intervention, an in-home intervention for community dwelling older adults with excessive FoF, incorporated the exposure as a more elementary part of the program [29]. To the best of our knowledge, based on the intervention protocols, all programs had comparable frequency of delivery for psychoeducation on home safety and relapse prevention. Comparable to our program, the ABLE program included psychoeducation on the background on anxiety consequences and rationale for treatment [28, 29, 33]. The only other treatment program for FoF in this specific target group, the *‘Step by Step intervention’* includes problem-solving and relaxation techniques as additional items as compared to the FIT-HIP intervention [33]. The intended therapy intensity of cognitive therapy in this program was similar to our intervention. Hence, the therapy intensity of the individual FIT-HIP intervention items, in the form of *therapy frequency*, does in itself not clearly explain the lack of effectivity of the FIT-HIP intervention.

Regarding the feasibility of the telephonic booster (6 weeks after discharge): this element proved to be easily forgotten, as the physiotherapist was no longer involved in the patient’s treatment after discharge. We incorporated the booster in the intervention based on lessons learned from the programs based on a ‘*Matter of Balance* ‘ [30, 34], and the insight that (increase in) FoF is common after discharge from geriatric rehabilitation [35]. We can, however, question whether a telephonic booster is useful for our target group, as patients who received the booster reported no benefit from this intervention element. Perhaps it would be more appropriate to extend the treatment for FoF to an ambulatory rehabilitation setting (in-home) [27, 36].

An important barrier to acknowledge is the limited level of the FoF reported after enrollment in the study (i.e. selection of the target population). Facilitators pointed out that during screening (first week of rehabilitation), patients were mainly sedentary. Once patients started the process of mobilization (i.e. walking during therapy), in clinical practice the FoF appeared to decrease. The timing of the intervention in relation to the timeline after fracture may be a relevant factor to consider in the selection of the target group. Current literature illustrates that FoF present 2–4 weeks after fracture is not associated with negative effects on long-term functional outcomes, contrary to FoF present 6–12 weeks post-fracture [7, 8]. Provided that the fear is not *disproportionate* and does not lead to significant avoidance behavior (activity restriction), this could imply that FoF shortly after fracture can in some cases be a normal or adaptive process which does not require treatment. Unfortunately, for this specific group of patients, it is currently unknown what a *disproportionate level* of FoF is as measured with established instruments such as the FES-I. We can question whether the standard cut-off values are appropriate for this target group, especially because the FES-I appears to be more closely related to functional performance than to psychological concepts such as anxiety [37]. Patients with hip fracture experience a sudden impairment of the lower body function, and a certain level of ‘caution’ in relation to an increased fall risk in the early stage of recovery after fracture, may be an appropriate response. For clinical practice it seems relevant to monitor the course of FoF. Findings from a cohort study of hip fracture patients show three distinct patterns of FoF evolving from 4 to 12 weeks after fracture; i] patients with consistently low levels of FoF; ii] patients with high levels of FoF at 4 weeks that continue to increase; iii] patients with high levels of FoF at 4 weeks which decrease at 12 weeks post-fracture [38]. It is currently unknown how these distinct trajectories relate to avoidance behavior. However, it is plausible that especially those patients that have increasing levels of FoF are more susceptible to develop activity restriction as a consequence of FoF. Accordingly this may be an important group to address by means of intervention.

Another factor to consider when screening for FoF, is the (mediating) role of anxiety (traits) in the development of maladaptive or dysfunctional fear of falling [18, 39, 40]. Findings from Bower et al. show that patients with higher scores for neuroticism were more likely to have high levels of FoF [38]. Also, the previously mentioned in-home cognitive behavioral program for FoF that was conducted by physiotherapists and showed positive effects on reducing FoF and activity restriction, was aimed at patients with *disproportionate* FoF; as defined as high fear and low to moderate objective fall risk and functional impairment because of FoF [29]. The majority of participants had a psychiatric disorder, most frequently a pre-existing anxiety disorder [18]. In contrast, the FIT-HIP study population reported low scores for anxiety, had a lower level of FoF at baseline (Falls Efficacy Score-International); and we excluded patients with generalized anxiety [12, 21]. It may therefore be useful to incorporate screening for more generalized anxiety symptoms and also specifically include patients with anxiety for treatment.

### Limitations

This process evaluation has several limitations. First, we cannot rule out the possibility of socially desirable answers given by patients and facilitators. To reduce the risk of such bias, we informed patients that data would be handled confidentially by the research team (not involved in treatment). For facilitators, we emphasized that their input was essential to improve the intervention for future purposes. Second, the timing of the interviews may have led to recall bias among facilitators and patients. However, facilitators had no trouble recalling the intervention and were able to identify barriers and suggest improvements. Additionally, we collected information on barriers from the regular informal contact with physiotherapists (researcher log) during the course of the study. We therefore have extensive information concerning the intervention’s feasibility, especially from the facilitator’s perspective. A third limitation is the relatively low response for the evaluation questionnaires from patients at discharge from rehabilitation. Physiotherapists coordinated this assessment, as the date of discharge could occasionally be brought forward. They sometimes forgot to hand out the questionnaires. Despite additional postal ‘follow-up’ in these cases, the response rate remained limited. Finally, data on performance according to protocol (including fidelity), was limited to self-report measures (session logs and interviews), which can lead to more favorable responses in comparison to more objective measures. However, video recording of the physiotherapy sessions was considered to be too intrusive for the patients. The strength of this process evaluation is that the results are based on extensive quantitative and qualitative information obtained from patients and facilitators (both physiotherapists and psychologists). This was analyzed within a well-established framework for process evaluations (Saunders) [22] and provided a good insight into the feasibility of the FIT-HIP intervention, possible barriers to implementation and suggestions for improving the intervention.

### Recommendations for improvement

First, in order to select an appropriate target population that can benefit from treatment, it is crucial to select patients with maladaptive FoF. Currently we do not know how to accurately quantify *disproportionate* levels of fear of falling for this specific target group. However, factors such as anxiety and avoidance behavior may contribute to the development of maladaptive FoF, and may aid the process of determining which patients require treatment. We therefore recommend screening patients for FoF, related activity restriction and comorbid anxiety at the start of the rehabilitation, and every time the rehabilitation treatment is evaluated. To assess activity restriction related to FoF, an instrument such as SAFE (Survey of Activities and Fear of Falling in the Elderly) could prove to be useful [41]. Treatment of FoF does not by definition have to be initiated directly at the start of rehabilitation, but treatment is advised when avoidance behavior for physical activities is observed. We also recommend treatment for FoF in the event the FoF is progressive or persists, which implies treatment in later stages of rehabilitation.

Second, to improve the feasibility of the FIT-HIP intervention we recommend the following adjustments regarding the content and organization of the intervention. 1] Intensify collaboration between physiotherapists and psychologists to (a form of) collective treatment, in order to support performance feedback for the physiotherapists and to enable timely identification when treatment is required from a psychologist. We advise that psychologists observe the patient during a physiotherapy session once a week. Furthermore, within each individual team, there should be clear agreements regarding the extent to which cognitive restructuring is provided by the physiotherapist (based on prior experience and the preferences of the physiotherapist), and which indications require referral to the psychologist. 2] We support the idea of a more tailored approach to applying guided exposure and cognitive restructuring. Based in part on the presence of anxiety traits, facilitators observed that some patients were more receptive to guided exposure and others to cognitive restructuring. We propose that physiotherapists continue to initiate treatment with both approaches and that the (most) appropriate treatment is determined during the joint treatment with psychologists. 3] More attention to cognitive restructuring in the training of facilitators may also be beneficial, as this element was perceived as most challenging. 4] Last, the telephonic booster can be eliminated from the intervention, due to lack of both feasibility for the facilitators and perceived benefit of the patients.

## Conclusion

This process evaluation shows that the FIT-HIP intervention was only partly feasible, which may have contributed to the lack of effectiveness of the intervention. To improve feasibility and effectiveness, we recommend a number of adjustments to the intervention. These include selecting patients with *maladaptive* FoF (specifically in the context of avoidance behavior for physical activities), being more flexible with regard to the timing of the intervention (initiating treatment at a later stage of rehabilitation), and providing more support to the physiotherapists with regard to the cognitive restructuring. Although the FIT-HIP intervention in its current form was not effective, and only partly feasible, there is sufficient evidence that cognitive behavioral therapy is a feasible and effective approach to reduce FoF in older adults. We therefore expect that, with the proposed improvements, the FIT-HIP intervention has the potential to effectively reduce FoF. However, further research is needed to prove whether the suggested adjustments result in improved feasibility and effectiveness of the intervention.

## Supplementary Information

**Additional file 1.** Topic list for psycho-education within the FIT-HIP intervention. This table provides an overview of the topics handled within the psycho-education of the FIT-HIP intervention.

**Additional file 2.** Enrollment of and data from patients and facilitators per GR unit. This table provides an overview of the enrollment and data available for each individual participating GR unit.

**Additional file 3.** Fear of falling and associated activity restriction. Table in word document (.doc). The table contains data on the course of fear of falling in the study population.

**Additional file 4.** Feedback and suggestions for improvement of the intervention provided by facilitators. This table provides an overview of additional (minor) suggestions for improvement of the intervention.

**Additional file 5.** Evaluation questionnaires. Summary of the evaluation questionnaires used in the process evaluation.

## Data Availability

The datasets used and/or analyzed during the current study are available from the corresponding author on reasonable request.

## Abbreviations

FES-I: Falls Efficacy Scale-International
FoF: Fear of Falling
FIT-HIP: Fear of falling InTervention in HIP fracture geriatric rehabilitation
LUMC: Leiden University Medical Center
