# Supplementary material for: Feasibility of a multicomponent cognitive behavioral intervention for fear of falling after hip fracture: process evaluation of the FIT-HIP intervention
Source: BMC Geriatr. 2021 Apr 1;21:224. doi: 10.1186/s12877-021-02170-5 (PMC8017759; doi:10.1186/s12877-021-02170-5)
Supplement: Supplementary file 1 — Additional file 1. Topic list for psycho-education within the FIT-HIP intervention. This table provides an overview of the topics handled within the psycho-education of the FIT-HIP intervention. [file 12877_2021_2170_MOESM1_ESM.docx]

**Additional file 1. Topic list for psychoeducation within the FIT-HIP intervention**

| Part | Main topic(s) | Subtopics |
| --- | --- | --- |
| 1 | Fear in general | Background on the function of fear. Dysfunctional forms of fear. Consequences of fear (on behavior); short term - relief of feelings of anxiety; long term - tendency to keep avoiding the situation. Leading to reduced self-efficacy. |
|  | Fear of falling | Background on fear of falling - definition, symptoms, prevalence.  Behavioral consequences: activity restriction; avoidance of physical activities with consequences for muscle strength, condition, balance. Increased risk of falling. Impact on social participation. Reduced quality of life. |
|  | Perspective | Treatment possibilities – a guided approach to help stay active. Practicing physical activity under supervision in a controllable manner. |
| 2 | Guided exposure | Background on behavioral therapy. Interaction between behavior, cognition (thoughts) and emotion (feelings). Behavior influences emotional state. Behavioral therapy addresses behavior; evaluating how to alter the behavior to more functional forms.  Background on guided exposure. Gradual, graded exposure to fearful situations: repeated mild anxiety response in controlled setting, eventually leading to reduction or extinction of fear.  FIT-HIP fear ladders, illustrating the ‘stepped’ approach to the graded exposure to feared situations. |
| 3 | Cognitive therapy | Background on cognitive therapy. Interaction between cognitions (thoughts), emotion (feelings) and behavior. Thoughts and/or cognitions can be affect how we feel. And this can in turn influence our behavior. Automatic thoughts.  Background on cognitive behavioral therapy: analyzing cognitions. Are they helpful? And if not, how can we address this to formulate more helpful cognitions. |
| 4 | General-prevention: *physical activity* | Stay active to keep muscles strong and supple. This will help reduce fall risk and in the event of accidental fall, aid in getting up easier. Thirty minutes of activity a day is helpful; for example walking, cycling, or house-hold chores. |
|  | General fall-prevention: *home-safety* | Awareness for: sufficient lighting, potential hazards in home (cables, rugs, doorsteps), sufficient passageway for walking with walking aids. Alert systems. Occupational therapy: home-safety evaluation |
|  | Fall-prevention: *other* | Awareness for: footwear, vision, medication |
| 5 | Personalized fall-prevention | Personalized advice regarding walking aids. Personalized advice regarding physical activity; with suggestions for exercises to perform. How to integrate exercise in the day schedule. Physical activity ‘buddy’. Personalized advice regarding home safety. |
| 6 | Relapse prevention | Involve significant others (friends, family) to help stay active (physical activity buddy). Personal advice on how to recognize a relapse (which behavior, which feelings, which non-helpful thoughts). Advice how to address the non-helpful thoughts  Ask help, discuss FoF with others (for example GP) |
