# Supplementary material for: Feasibility of a multicomponent cognitive behavioral intervention for fear of falling after hip fracture: process evaluation of the FIT-HIP intervention
Source: BMC Geriatr. 2021 Apr 1;21:224. doi: 10.1186/s12877-021-02170-5 (PMC8017759; doi:10.1186/s12877-021-02170-5)
Supplement: Supplementary file 2 — Additional file 2. Enrollment of and data from patients and facilitators per GR unit. This table provides an overview of the enrollment and data available for each individual participating GR unit. [file 12877_2021_2170_MOESM2_ESM.docx]

**Additional file 2. Enrollment of and data on patients and facilitators per Geriatric Rehabilitation unit**

|  | **All units** | **GR unit 1** | **GR unit 2** | **GR unit 3** | **GR unit 4** | **GR unit 5*** | **GR unit 6** |
| --- | --- | --- | --- | --- | --- | --- | --- |
| **Patients (n)** |  |  |  |  |  |  |  |
| Included in the study | 39 | 5 | 5 | 11 | 1 | 9 | 8 |
| Who received the intervention | 37 | 5 | 5 | 11 | 1 | 7 | 8 |
| With completed evaluation questionnaire at discharge | 20 | 3 | 3 | 5 | 0 | 3 | 6 |
| With completed evaluation questionnaire at 3-month follow up | 23 | 3 | 4 | 7 | 0 | 3 | 6 |
| With completed evaluation questionnaire at 6-month follow up | 23 | 4 | 4 | 6 | 0 | 4 | 5 |
| Participating in patient interviews | 9 | 2 | 1 | 3 | 0 | 1 | 2 |
| **Physiotherapists (n)** |  |  |  |  |  |  |  |
| Trained to conduct the FIT-HIP intervention | 14 | 2 | 2 | 2 | 2 | 4 | 2 |
| Completing study | 12 | 2 | 1 | 2 | 2 | 3 | 2 |
| Participating in evaluation interview | 10 | 2 | 1 | 2 | 0 | 3 | 2 |
| **Psychologists (n)** |  |  |  |  |  |  |  |
| Involved in the FIT-HIP intervention | 8 | 1 | 1 | 1 | 1 | 3 | 1 |
| Completing study | 7 | 1 | 1 | 0 | 1 | 3 | 1 |
| Participating in evaluation interview | 6 | 1 | 1 | 0 | 1 | 2 | 1 |

**Note**: * Geriatric Rehabilitation unit with a co-location included and trained 4 months after start of the trial.
