# Supplementary material for: Feasibility of a multicomponent cognitive behavioral intervention for fear of falling after hip fracture: process evaluation of the FIT-HIP intervention
Source: BMC Geriatr. 2021 Apr 1;21:224. doi: 10.1186/s12877-021-02170-5 (PMC8017759; doi:10.1186/s12877-021-02170-5)
Supplement: Supplementary file 3 — Additional file 3. Fear of falling and associated activity restriction. Table in word document (.doc). The table contains data on the course of fear of falling in the study population. [file 12877_2021_2170_MOESM3_ESM.docx]

**Additional file 3. Fear of falling and associated activity restriction**

|  | **Baseline**  (n=39) | **Discharge** (n=34) | **3 month FU** (n=24) | **6 month FU**  (n=25) |
| --- | --- | --- | --- | --- |
| **Falls-Efficacy Scale International (FES-I) total score***  **Range 0-64; mean (SD)** | 33.9 (9.9) | 32.8 (11.0) | 35.1 (13.9) | 36.5 (12.1) |
|  |  |  |  |  |
| **Level of fear of falling measured with the VAS-score**†*****  **Range 0-100; mean (SD)** | 54.0 (17.4) | 46.3 (24.2) | 52.1 (28.8) | 48.6 (28.1) |
|  |  |  |  |  |
| **Fear of falling measured with the 1-item question**‡  *‘Are you concerned to fall?’* |  |  |  |  |
| **Number of participants with this response (%)** |  |  | ** | ** |
| Never | 0 | 3 (8.8) | 0 | 1 (4.2) |
| Almost never | 3 (7.7) | 6 (17.6) | 3 (13.0) | 3 (12.5) |
| Sometimes | 24 (61.5) | 17 (50.0) | 10 (43.5) | 12 (50.0) |
| Often | 10 (25.6) | 6 (17.6) | 6 (26.1) | 5 (20.8) |
| Very often | 2 (5.1) | 2 (5.9) | 4 (17.4) | 3 (12.5) |
|  |  |  |  |  |
| **Activity restriction measured with the 1-item question**‡**;**  *‘Do you avoid activities due to fear of falling?’* |  |  |  |  |
| **Number of participants with this response (%)** |  |  | ** | ** |
| Never | § | 21 (61.8) | 3 (13.0) | 6 (25.0) |
| Almost never | § | 7 (20.6) | 6 (26.1) | 5 (20.8) |
| Sometimes | § | 5 (14.7) | 7 (30.4) | 9 (37.5) |
| Often | § | 1 (2.9) | 4 (17.4) | 3 (12.5) |
| Very often | § | 0 | 3 (13.0) | 1 (4.2) |
|  |  |  |  |  |

**Notes**: FU= follow up. *Lower scores indicate less fear of falling. †VAS = Visual analogue scale. VAS-FoF: ‘*On a scale of 0-100, with 0 being no concerns and 100 exceptionally high levels of concerns about falling, how would you rate your concern about falling?’* ‡ Based on a 5-point Likert scale with answer categories: *never; almost never; sometimes; often; very often*. § Not applicable **Numbers do not add up to final numbers due to missing data.
