# Supplementary material for: Feasibility of a multicomponent cognitive behavioral intervention for fear of falling after hip fracture: process evaluation of the FIT-HIP intervention
Source: BMC Geriatr. 2021 Apr 1;21:224. doi: 10.1186/s12877-021-02170-5 (PMC8017759; doi:10.1186/s12877-021-02170-5)
Supplement: Supplementary file 4 — Additional file 4. Feedback and suggestions for improvement of the intervention provided by facilitators. This table provides an overview of additional (minor) suggestions for improvement of the intervention. [file 12877_2021_2170_MOESM4_ESM.docx]

| **Additional file 4. Feedback and suggestions for improvement of the intervention provided by facilitators** | | |
| --- | --- | --- |
| **Intervention element** | **Feedback** | **Suggestions for improvement** |
| FIT-HIP intervention  (as a whole) | 1] Limited level of fear of falling (FoF) was perceived as a barrier to performing the intervention. | 1 a] Improve the assessment of FoF, to determine those forms that require treatment (maladaptive FoF).  1 b] Consider starting treatment at a later stage of the inpatient rehabilitation. |
| FIT-HIP intake | 1] Patients brought up few goals regarding (social) participation. | 1] - |
|  | 2] In the current format, insight into the coping strategies used by patients is lacking | 2] Consider adding the concept of illness beliefs to the intake. This provides insight into coping strategies. |
| Guided exposure | 1] Patients may experience difficulty in formulating goals for fear ladders, due to cognitive impairment or lack of practical insight and understanding of the recovery process and rehabilitation goals. | 1] Facilitators may need to provide (more) assistance in formulating goals for the fear ladders. |
|  | 2] Limited level of FoF can be a barrier to employing guided exposure. | 2] Use fear ladders/guided exposure on indication.  (tailoring intervention) |
|  | 3] The use of fear ladders for patients with more generalized forms of anxiety may enhance their fear and therefore be less appropriate. | 3] Use fear ladders/guided exposure on indication.  (tailoring intervention) |
|  | 4] It can be challenging to involve the entire health care team to support the guided exposure. | 4] Involve the nursing staff and physician in drawing up the treatment plans for guided exposure. |
| Psychoeducation | 1] Time and (re)sources to embed psychoeducation in care as usual are limited and  can be perceived as a barrier to conducting it. | 1 a] Consider embedding the psychoeducation into group sessions (with other target groups than hip fracture patients).  1 b] Consider a handout with information instead of psychoeducation provided by physiotherapist. |
|  | 2] Patients and other health care professionals may have different expectations regarding the content of physical therapy sessions (i.e. more physical exercises, less cognitive therapy). | 2] The facilitators who perceive patients’ expectation of the physiotherapist’s role to be a barrier, suggest that psychoeducation be provided by a psychologist. |
| Cognitive restructuring including homework | 1] Limited level of FoF can be a barrier to conducting the cognitive restructuring. | 1] Use cognitive restructuring on indication (tailoring intervention). |
|  | 2] Cognitive restructuring can be time-consuming due to limited experience of the physiotherapist. | 2] Perform cognitive restructuring together with psychologist (mentoring), to gain more experience. |
|  | 3] Cognitive restructuring can be difficult to perform. Not a role for physiotherapists. | 3] The physiotherapists who state that cognitive restructuring is not part of a physiotherapist’s role/work, suggest that cognitive restructuring is performed by psychologists. |
|  | 4] Patients and other health care professionals may have different expectations regarding the content of physical therapy sessions (i.e. more physical exercises, less cognitive therapy). | 4] Those facilitators who perceive patients’ expectation of the physiotherapist’s role to be a barrier, suggest the cognitive restructuring be conducted by a psychologist. |
|  | 5] One physiotherapist mentioned that the template for cognitive restructuring is difficult to use in this target group. | 5] Simplify the template for cognitive restructuring. |
|  | 6] Cognitive impairment can make cognitive restructuring more challenging to perform. | 6] Short-term effects (during the therapy session) can still be achieved. Application of cognitive restructuring can therefore still be appropriate. |
| Staying Active Plan | 1] All physiotherapists questioned the long-term benefit of the Staying Active Plan. | 1] - |
|  | 2] There is a limited input/contribution from the patient (regarding personalized goals). | 2] Facilitators may need to provide (more) assistance in formulating personalized goals. |
|  | 3] It is difficult to involve informal care givers (often children who work). | 3] - |
| Telephonic booster | 1] If problems occur after discharge, they will be present soon after discharge. | 1] Perform the booster shortly after discharge. |
|  | 2] Reimbursement for inpatient geriatric rehabilitation stops after discharge. Therefore, there is no financing for the booster. | 2] - |
| Motivational interviewing | 1] Some facilitators had limited prior experience with motivational interviewing. | 1] Provide additional training to facilitators with limited experience in motivational interviewing. |
