# Supplementary material for: Feasibility of a multicomponent cognitive behavioral intervention for fear of falling after hip fracture: process evaluation of the FIT-HIP intervention
Source: BMC Geriatr. 2021 Apr 1;21:224. doi: 10.1186/s12877-021-02170-5 (PMC8017759; doi:10.1186/s12877-021-02170-5)
Supplement: Supplementary file 5 — Additional file 5. Evaluation questionnaires. Summary of the evaluation questionnaires used in the process evaluation. [file 12877_2021_2170_MOESM5_ESM.docx]

**Additional file 5. Evaluation questionnaires (English)**

Seven different evaluation questionnaires were used to assess feasibility of the FIT-HIP intervention for patients, facilitators (physiotherapists and psychologists) and other health-care professionals in geriatric rehabilitation (nursing staff, elderly care physician). The questionnaires have been translated from the original language (Dutch) to English. The questionnaires are summarized below.

**Questionnaire 1**

**Patient evaluation questionnaire – T1. Discharge from inpatient geriatric rehabilitation**

**Background**

In the past few weeks you have participated in the FIT-HIP trial, a study aimed at evaluating the treatment of fear of falling after hip fracture. The inpatient rehabilitation treatment program you were following has recently ended (or will soon finish). We are interested in your experience with the treatment for fear of falling provided within the rehabilitation program. We therefore kindly ask you to answer the following questions. In this questionnaire we focus on treatment provided by physiotherapists and, if applicable, psychologists.

The data will be handled confidentially, only the research team has insight into this information (the therapists do not have insight into your answers).

**Physiotherapy**

1. On a scale of 1-10, how satisfied are you with the treatment provided to you during the physiotherapy sessions?

- I would rate the physiotherapy sessions: … [0-10]

1. What is your general opinion about the (quality of) the physiotherapist(s)?

- Very poor
- Poor
- Sufficient / average
- Good
- Very good

1. Was the physiotherapy treatment helpful to reduce the level of fear of falling?

- No, not at all
- No, barely
- Yes, a little
- Yes, a lot
- Yes, very much

1. What do you think of the physical effort expected of you during physical therapy?

- Far too much
- Too much
- Just right
- Not (quite) enough
- Not nearly enough

1. Was the following content of physiotherapy treatment helpful to reduce the level of fear of falling?
2. Information about fear of falling and fall-risk

- No, not at all
- No, barely
- Yes, a little
- Yes, a lot
- Yes, very much
- Not applicable

1. Guided exposure to physical activity, based on your FIT-HIP treatment plan

- No, not at all
- No, barely
- Yes, a little
- Yes, a lot
- Yes, very much
- Not applicable

1. Challenging your thoughts about falling

- No, not at all
- No, barely
- Yes, a little
- Yes, a lot
- Yes, very much
- Not applicable

1. Home work: physical exercise

- No, not at all
- No, barely
- Yes, a little
- Yes, a lot
- Yes, very much
- Not applicable

1. Home work: challenging thoughts about falling

- No, not at all
- No, barely
- Yes, a little
- Yes, a lot
- Yes, very much
- Not applicable

1. Composing the ‘Staying Active Plan’ with your therapist

- No, not at all
- No, barely
- Yes, a little
- Yes, a lot
- Yes, very much
- Not applicable

1. Did you do the homework (physical exercises) provided to you?

- Never
- Seldom
- Sometimes
- Usually
- Always

1. On average, per week, how much time did you spend on the homework (physical exercises) [….minutes] / week
2. Did you use the worksheet ‘*Challenging Thoughts*’ for homework assignment(s)?

- No
- Yes, I used this worksheet [….] time(s)

**Treatment provided by a psychologist**

1. Did you receive treatment from a psychologist, specifically for the fear of falling?

- No 🡪 you may continue with question 13
- Yes 🡪 you may continue with question 10

1. On a scale of 1-10, how satisfied are you with the treatment provided to you by the psychologist?

- I would rate the treatment provided by the psychologist: … [0-10]

1. What is your general opinion about the (quality of) the psychologist?

- Very poor
- Poor
- Sufficient / average
- Good
- Very good

12. Was the treatment provided by the psychologist helpful to reduce the level of fear of falling?

- No, not at all
- No, barely
- Yes, a little
- Yes, a lot
- Yes, very much

**Inpatient rehabilitation treatment program**

1. Would you recommend this treatment program for fear of falling, provided within the inpatient rehabilitation, to friends or family?

- No
- Yes

1. Do you have any additional remarks regarding the treatment program?

**Questionnaire 2**

**Patient evaluation questionnaire – T2. Three months after discharge from geriatric rehabilitation**

**Background**

In the past few months you have participated in the FIT-HIP trial, a study aimed at evaluating the treatment of fear of falling after hip fracture. Three months ago you were discharged from inpatient geriatric rehabilitation. We are interested in the perceived benefit of the treatment program to reduce fear of falling, specifically after discharge home. We therefore kindly ask you to answer the following questions.

**Rehabilitation treatment program**

1. Was the inpatient geriatric rehabilitation treatment program helpful to reduce the level of fear of falling in the past three months?

- No, not at all
- No, barely
- Yes, a little
- Yes, a lot
- Yes, very much

1. As a result of the inpatient geriatric rehabilitation treatment program, in the past three months,:
   1. I am less concerned to fall
      - Disagree
      - Agree
   2. I have avoided less activities
      - Disagree
      - Agree

**Telephonic consultation with physiotherapist**

1. Was the telephonic consultation with the physiotherapist, a few weeks after discharge from inpatient geriatric rehabilitation, helpful to reduce the level of fear of falling?

- No, not at all
- No, barely
- Yes, a little
- Yes, a lot
- Yes, very much
- I did not receive a telephonic consultation with the physiotherapist

**Staying Active Plan**

1. Has your ‘*Staying Active Plan’* been helpful to reduce the level of fear of falling in the past three months?

- No, not at all
- No, barely
- Yes, a little
- Yes, a lot
- Yes, very much
- I do not have a ‘*Staying Active Plan’*

1. Your ‘*Staying Active Plan’* contains suggestions for physical exercises. Have you practiced these suggested exercises in the past three months?

- Never
- Seldom
- Sometimes
- Usually
- Always

1. Your ‘*Staying Active Plan’* discusses situations which can trigger fear of falling, and gives suggestions what can be helpful to do in such circumstances. Have the suggestions been helpful to reduce the level of fear of falling in the past three months?

- No, not at all
- No, barely
- Yes, a little
- Yes, a lot
- Yes, very much
- I do not have a ‘*Staying Active Plan’*

1. Do you have any additional remarks?

**Questionnaire 3**

**Patient evaluation questionnaire – T3. Six months after discharge from geriatric rehabilitation**

**Background**

In the past few months you have participated in the FIT-HIP trial, a study aimed at evaluating the treatment of fear of falling after hip fracture. Six months ago you were discharged from inpatient geriatric rehabilitation. We are interested in the perceived benefit of the treatment program to reduce fear of falling, specifically after discharge home. We therefore kindly ask you to answer the following questions.

**Rehabilitation treatment program**

1. Was the inpatient geriatric rehabilitation treatment program helpful to reduce the level of fear of falling in the past three months?

- No, not at all
- No, barely
- Yes, a little
- Yes, a lot
- Yes, very much

1. As a result of the inpatient geriatric rehabilitation treatment program, in the past three months:
   1. I am less concerned to fall
      - Disagree
      - Agree
   2. I have avoided less activities
      - Disagree
      - Agree

**Staying Active Plan**

1. Has your ‘*Staying Active Plan’* been helpful to reduce the level of fear of falling in the past three months?

- No, not at all
- No, barely
- Yes, a little
- Yes, a lot
- Yes, very much
- I do not have a ‘*Staying Active Plan’*

1. Your ‘*Staying Active Plan’* contains suggestions for physical exercises. Have you practiced these suggested exercises in the past three months?

- Never
- Seldom
- Sometimes
- Usually
- Always

1. Your ‘*Staying Active Plan’* discusses situations which can trigger fear of falling, and gives suggestions what can be helpful to do in such circumstances. Have the suggestions been helpful to reduce the level of fear of falling in the past three months?

- No, not at all
- No, barely
- Yes, a little
- Yes, a lot
- Yes, very much
- I do not have a ‘*Staying Active Plan’*

1. Do you have any additional remarks?

**Questionnaire 4**

**Evaluation questionnaire FIT-HIP intervention – physiotherapist(s)**

**Background**

The FIT-HIP trial, aimed at evaluating the treatment of fear of falling after hip fracture, has been performed within your health care organization. In the past few months, you have conducted the FIT-HIP intervention. We are interested in your experience with the intervention, and would like to gain insight into the feasibility of the intervention. We therefore kindly ask you to answer the following questions.

**General opinion of the FIT-HIP intervention**

1. On a scale of 1-10, what is your general opinion of the FIT-HIP intervention?

- I would rate the FIT-HIP intervention: … [0-10]

**Feasibility of the FIT-HIP intervention**

1. To what extent were you able to adequately apply the following elements of the FIT-HIP intervention?
   1. Provide psycho-education (concerning fear of falling and fall-risk)

- Insufficiently
- Barely
- Reasonably
- Well
- Very well
  1. Perform the FIT-HIP intake interview
- Insufficiently
- Barely
- Reasonably
- Well
- Very well
  1. Compose the individual FIT-HIP treatment plan (FIT-HIP fear ladders)
- Insufficiently
- Barely
- Reasonably
- Well
- Very well
  1. Guided exposure (using the individual FIT-HIP treatment plan)
- Insufficiently
- Barely
- Reasonably
- Well
- Very well
  1. Assess fear of falling with VAS-scale
- Insufficiently
- Barely
- Reasonably
- Well
- Very well
  1. Cognitive restructuring (challenging thoughts)
- Insufficiently
- Barely
- Reasonably
- Well
- Very well
  1. Motivational interviewing techniques
- Insufficiently
- Barely
- Reasonably
- Well
- Very well
  1. Compose the ‘Staying Active Plan’
- Insufficiently
- Barely
- Reasonably
- Well
- Very well
  1. Conduct the telephonic consultation (booster after discharge)
- Insufficiently
- Barely
- Reasonably
- Well
- Very well

1. Is time-constraint a barrier to any future application of the FIT-HIP intervention ?

- No
- Yes

**Perceived benefit of the FIT-HIP intervention**

1. In your opinion, have patients had benefit of the FIT-HIP intervention?

- No, not at all
- No, barely
- Yes, a little
- Yes, a lot
- Yes, very much

1. In your opinion, have patients had benefit of the following elements of FIT-HIP intervention?
   1. Psycho-education (concerning fear of falling and fall-risk)

- No, not at all
- No, barely
- Yes, a little
- Yes, a lot
- Yes, very much
  1. Guided exposure
- No, not at all
- No, barely
- Yes, a little
- Yes, a lot
- Yes, very much
  1. Cognitive restructuring (challenging thoughts) regarding (fear of) falling
- No, not at all
- No, barely
- Yes, a little
- Yes, a lot
- Yes, very much
  1. Motivational interviewing techniques
- No, not at all
- No, barely
- Yes, a little
- Yes, a lot
- Yes, very much
  1. The ‘Staying Active Plan’
- No, not at all
- No, barely
- Yes, a little
- Yes, a lot
- Yes, very much
  1. Telephonic consultation (booster after discharge)
- No, not at all
- No, barely
- Yes, a little
- Yes, a lot
- Yes, very much

**Specific instruments and methods**

1. Was the use of the Goal Attainment Scale helpful?

- No, not at all
- Yes, a little
- Yes, very

1. Was the use of the VAS-scale for assessing fear of falling during the guided exposure helpful?

- No, not at all
- Yes, a little
- Yes, very

1. Was the use of the worksheet *‘Challenging Thoughts’* helpful for the patients?

- No, not at all
- Yes, a little
- Yes, very

1. Was the monthly coaching with psychologists helpful?

- No, not at all
- Yes, a little
- Yes, very

1. Did you have sufficient material to perform the FIT-HIP intervention. If not, please could you explain.

- Yes,
- No, namely…[….]

**Suggestions for improvement**

1. Do you have suggestions for improvement for the following elements of the FIT-HIP intervention:
2. Psycho-education (concerning fear of falling and fall-risk)
3. FIT-HIP intake interview
4. The individual FIT-HIP treatment plan (*FIT-HIP fear ladders*)
5. Guided exposure (using the *individual FIT-HIP treatment plan*)
6. Assessment of level of fear of falling with VAS-scale
7. Cognitive restructuring (*challenging thoughts*)
8. Motivational interviewing techniques
9. The ‘*Staying Active Plan’*
10. The telephonic consultation (booster after discharge)
11. Do you have additional remarks, or ideas to improve the intervention?

**Questionnaire 5**

**Evaluation questionnaire FIT-HIP intervention – psychologist**

**Background**

The FIT-HIP trial, aimed at evaluating the treatment of fear of falling after hip fracture, has been performed within your health care organization. In the past few months you have been involved in the FIT-HIP intervention. We are interested in your experience with the intervention, and would like to gain insight into the feasibility of the intervention. Therefore we kindly request you to answer the following questions.

**The role of psychologist within the FIT-HIP intervention**

1. Have you, in the context of the FIT-HIP trial:
   1. Coached physiotherapist(s)
      - No
      - Yes
   2. Treated FIT-HIP patients for fear of falling
      - No
      - Yes
2. If you did provide treatment for fear of falling to FIT-HIP patients, what was the content of the therapy:
   1. Psycho-education
   2. Cognitive restructuring
   3. Guided exposure
   4. Other, namely …[….]
3. In your opinion, was the monthly coaching session helpful for the physiotherapists

- No, not at all
- Yes, a little
- Yes, very

**Feasibility of the FIT-HIP intervention**

1. In your opinion, are there certain elements of the FIT-HIP intervention that are challenging for a physiotherapist to perform. What are possible reasons for this?

- No
- Yes, namely:
  - Psycho-education …[….]
  - Guided exposure …[….]
  - Other, namely …[….]

1. In your experience, do patients with fear of falling have specific characteristics, which would indicate treatment by a psychologist (in addition to or instead of a physiotherapist)
   - No,
   - Yes, namely …[….]

**General opinion of the FIT-HIP intervention**

1. On a scale of 1-10, what is your general opinion of the FIT-HIP intervention?

- I would rate the FIT-HIP intervention: … [0-10]

**Perceived benefit of the FIT-HIP intervention**

1. In your opinion, have patients had benefit of the following elements of FIT-HIP intervention, provided by physiotherapists?
2. Psycho-education (concerning fear of falling and fall-risk)

- No, not at all
- No, barely
- Yes, a little
- Yes, a lot
- Yes, very much
- I don’t know

1. Guided exposure

- No, not at all
- No, barely
- Yes, a little
- Yes, a lot
- Yes, very much
- I don’t know

1. Cognitive restructuring (challenging thoughts) regarding (fear of) falling

- No, not at all
- No, barely
- Yes, a little
- Yes, a lot
- Yes, very much
- I don’t know

1. Motivational interviewing techniques

- No, not at all
- No, barely
- Yes, a little
- Yes, a lot
- Yes, very much
- I don’t know

1. The ‘Staying Active Plan’

- No, not at all
- No, barely
- Yes, a little
- Yes, a lot
- Yes, very much
- I don’t know

1. Telephonic consultation (booster after discharge)

- No, not at all
- No, barely
- Yes, a little
- Yes, a lot
- Yes, very much
- I don’t know

**Suggestions for improvement**

1. Do you have suggestions for improvement for the following elements of the FIT-HIP intervention:
2. Guided exposure (using the individual FIT-HIP treatment plan)
3. Cognitive restructuring (challenging thoughts)
4. Motivational interviewing techniques
5. Do you have additional remarks, or ideas to improve the intervention?

**Questionnaire 6**

**Evaluation questionnaire FIT-HIP intervention – Elderly Care Physician**

**Background**

The FIT-HIP trial, aimed at evaluating the treatment of fear of falling after hip fracture, has been performed within your health care organization. In the past few months your patients (with hip fracture and fear of falling) have received the FIT-HIP intervention.

We would like to gain further insight into the feasibility of the intervention, for patients and health-care professionals. Therefore we kindly request you to answer the following questions.

**Individual FIT-HIP treatment plan**

1. Were you informed about the content of the individual FIT-HIP treatment plans?

- For all the FIT-HIP patients
- For the majority of the FIT-HIP patients
- For about half of the FIT-HIP patients
- For the minority of the FIT-HIP patients
- For none of the FIT-HIP participants patients

1. In your opinion, did the whole team of rehabilitation professionals adhere to/ follow the individual FIT-HIP treatment plan?

- Always
- Usually
- Sometimes
- Barely
- Never

**Perceived benefit of the FIT-HIP intervention**

1. In your opinion, have patients had benefit of the FIT-HIP intervention?

- No, not at all
- No, barely
- Yes, a little
- Yes, a lot
- Yes, very much

**General opinion of the FIT-HIP intervention**

1. On a scale of 1-10, what is your general opinion of the FIT-HIP intervention?

- I would rate the FIT-HIP intervention: … [0-10]

**Suggestions for improvement**

1. Do you have additional remarks, or ideas to improve the intervention?

**Questionnaire 7**

**Evaluation questionnaire FIT-HIP intervention – Nursing Staff**

**Background**

The FIT-HIP trial, aimed at evaluating the treatment of fear of falling after hip fracture, has been performed within your health care organization. In the past few months your patients (with hip fracture and fear of falling) have received the FIT-HIP intervention.

We would like to gain further insight into the feasibility of the intervention, for patients and health-care professionals. Therefore we kindly request you to answer the following questions.

**Individual FIT-HIP treatment plan**

1. Were you informed about the content of the individual FIT-HIP treatment plans?

- For all the FIT-HIP participants
- For the majority of the FIT-HIP participants
- For about the half of the FIT-HIP participants
- For the minority of the FIT-HIP participants
- For none of the FIT-HIP participants

1. How often were changes to the individual FIT-HIP treatment plans or progress discussed with the nursing staff (by the physiotherapist)?

- Every day
- Several times a week
- Once a week
- Once every two weeks
- Once a month
- Never

1. Did the nursing staff adhere to/ follow the individual treatment plans?

- Always
- Usually
- Sometimes
- Barely
- Never

**Perceived benefit of the FIT-HIP intervention**

1. In your opinion, have patients had benefit of the FIT-HIP intervention?

- No, not at all
- No, barely
- Yes, a little
- Yes, a lot
- Yes, very much

**General opinion of the FIT-HIP intervention**

1. On a scale of 1-10, what is your general opinion of the FIT-HIP intervention?

- I would rate the FIT-HIP intervention: … [0-10]

**Suggestions for improvement**

1. Do you have additional remarks, or ideas to improve the intervention?
